# Motif-designed peptide nanofibers as bioactive surface modifiers for biomimetic hydroxyapatite formation on demineralized porous dentin

**DOI:** 10.1039/d6ra00721j

**Published:** 2026-04-07

**Authors:** Xinyi Liu, Yingli Li, Wenting He, Mengyu Jiao, Yun Zhang, Chenxi Du, Gang Wei, Keqing Pan

**Affiliations:** a Department of Stomatology, The Affiliated Hospital of Qingdao University Qingdao 266003 China pankeqing77@qdu.edu.cn; b School of Stomatology, Qingdao University Qingdao 266023 China; c School of Polymer Science and Engineering, Qingdao University of Science and Technology Qingdao 266042 China duchenxi5@outlook.com wei@uni-bremen.de +86-15066242101

## Abstract

Dentin hypersensitivity (DH) is closely associated with mineral loss and subsequent exposure of dentinal tubules, which underscores the need for biomimetic strategies capable of restoring dentin as an organic–inorganic composite. In this work, a short peptide (KLVFFAKMLPHHGA) was designed by integrating a self-assembly motif with a mineralization-inducing segment, aiming to promote dentin remineralization. The peptide readily formed stable nanofibrous assemblies in ethanol containing 0.1% (v/v) trifluoroacetic acid (TFA; TFA : ethanol = 1 : 9) at 37 °C. These peptide nanofibers (PNFs) could be immobilized onto the demineralized dentin surface and acted as organic frameworks for calcium phosphate deposition. After *in vitro* remineralization under 1.5× simulated body fluid (1.5× SBF), a continuous and dense remineralized layer was gradually formed on the dentin surface. As the mineralization process progressed, mineral deposition extended inward along the dentinal tubules, resulting in effective deep tubule occlusion. Micromorphological observations and compositional analyses further revealed that the newly formed mineral phase exhibited structural and chemical features consistent with an apatite-like calcium phosphate phase. Compared with the control group, dentin treated with PNFs showed an improvement in surface microhardness. Meanwhile, biocompatibility evaluations indicated that the system exhibited favorable biocompatibility under the experimental conditions. Based on these findings, the self-assembling short PNF-mediated biomimetic mineralization strategy proposed in this study provides a facile materials design approach with potential applicability for the remineralization and functional improvement of demineralized dentin.

## Abstract 2

Motif-designed peptide nanofibers as bioactive surface modifiers promote biomimetic mineralization of hydroxyapatite on demineralized porous dentin materials.

## Graphical abstract

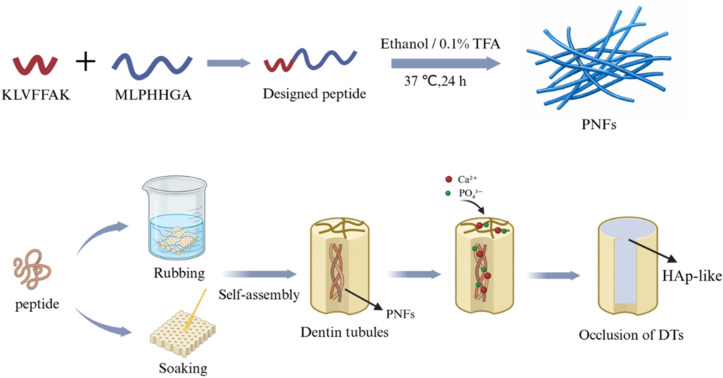

## 1 Introduction

Dentin hypersensitivity (DH) is frequently encountered in clinical practice and is typically experienced as brief, sharp pain elicited by thermal, chemical, or mechanical stimuli. Such discomfort can interfere with routine oral activities, including eating and tooth brushing, and consequently impair patients' quality of life.^1,2^ Population-based investigations and systematic analyses have shown that DH is not confined to specific patient groups and remains prevalent among young and middle-aged individuals. In most cases, its occurrence is closely associated with dentin exposure and the opening of dentinal tubules.^1–3^ From a structural perspective, dentin is not a simple mineralized tissue but a hierarchically organized organic–inorganic composite, in which the collagen matrix and mineral phase act cooperatively to maintain its mechanical integrity and functional performance. Once this finely balanced architecture is disrupted, therapeutic approaches that rely solely on superficial tubule sealing or conventional desensitizing agents often struggle to achieve long-lasting efficacy in the dynamic oral environment. As a result, recent efforts have increasingly shifted away from “simple occlusion” toward strategies aimed at structural repair, with the goal of restoring dentin mineral content and microstructural organization in a more stable manner. Within this context, biomimetic remineralization that is designed to emulate natural mineralization processes has attracted growing attention as a promising restorative approach.^3–5^

The fundamental framework of dentin consists of type I collagen fibrils, while the inorganic phase is dominated by hydroxyapatite (HAp), which is distributed both extrafibrillarly and intrafibrillarly, resulting in a coordinated mineralization pattern.^5–7^ Mineral dissolution and exposure of the collagen network induced by acid erosion or carious processes not only trigger hypersensitivity symptoms but also compromise the long-term stability of the resin–dentin interface.^3,4,8^ Accordingly, reconstructing the collagen–HAp composite architecture at the microscale and nanoscale to restore functional mineralization of dentin are regarded as major challenges in the prevention and management of DH.^3–5^

During natural mineralization, non-collagenous proteins (NCP) regulate the accumulation and transformation of Ca^2+^ and PO_4_^3−^ ions *via* charged or coordinative side chains, thereby playing a critical role in ordered mineral deposition both within and around collagen fibrils.^5,9^ Inspired by this mechanism, a range of biomimetic systems employ NCP analogues to facilitate the infiltration of amorphous calcium phosphate (ACP) into collagen networks in a precursor state, followed by its conversion into HAp under localized environmental regulation, thereby enabling bottom–up remineralization.^10–14^ Among these approaches, polymer-induced liquid precursor systems can markedly enhance mineralization depth and dentinal tubule occlusion, however, the intrinsic randomness of polymer architectures constrains precise spatial and directional control over mineral deposition.^10,15,16^ By contrast, proteins and peptide-based regulators, owing to their sequence programmability and precisely defined functional motifs, have emerged as focal components in biomimetic mineralization research.^17^ A variety of NCP-mimetic peptides, including functional fragments derived from CPP, DMP1 and DPP, have been demonstrated to stabilize ACP and regulate HAp nucleation and growth, thereby exhibiting distinct advantages in dentin remineralization.^9,18,19^ In addition, mineralization-directing peptides inspired by amelogenin templates, such as sADP5 and TRAP, can generate infiltrative mineralized layers on demineralized dentin surfaces, enabling simultaneous restoration of the surface and peritubular regions.^13,14^

Recently, peptide-based nanomaterials assembled through the combination of distinct functional segments have gained increasing attention, as they can be rationally designed to fulfill specific mineralization or self-assembly functions.^17^ For example, the bifunctional peptides CYP and DGP, which simultaneously incorporate substrate-anchoring motifs and mineralization-regulating sequences, have been shown to enhance both the depth of remineralization and the uniformity of mineral deposition in demineralized dentin.^20,21^ Another important class comprises self-assembling peptides, which can spontaneously organize into nanofibrous structures or hydrogels, thereby providing three-dimensional scaffolds for ion transport and mineral deposition.^17,22,23^ However, existing systems commonly suffer from insufficient interfacial binding stability or limited capability in regulating the ACP-to-HAp transformation.^20,24^

In light of the above considerations, a bifunctional peptide, KLVFFAKMLPHHGA, incorporating both a self-assembly module and a mineralization-inducing module, was designed. The KLVFFAK segment is capable of forming stable nanofibrous networks in aqueous environments, providing collagen-mimetic three-dimensional scaffolds on demineralized dentin surfaces and within dentinal tubules.^25–27^ The MLPHHGA segment is enriched in histidine residues capable of coordinating with Ca^2+^, which is expected to stabilize ACP precursors and direct their transformation toward HAp.^28^ Building on this dual-module design, the peptide was systematically evaluated in an *in vitro* demineralized dentin model, with particular attention to its self-assembly behavior, the ability of peptide nanofibers (PNFs) to regulate mineral formation, the resulting depth of dentinal tubule occlusion and biocompatibility, thereby offering a potential framework for the development of dentin remineralization materials that integrate structural biomimicry with precise mineralization control.

## 2 Materials and methods

### 2.1 Materials

The peptide KLVFFAKMLPHHGA (purity ≥ 95%) was provided by Jietai Biotechnology Co., Ltd (Nanjing, China). Ethanol, trifluoroacetic acid (TFA), NaCl, NaHCO_3_, KCl, K_2_HPO_4_, MgCl_2_, HCl, CaCl_2_, Na_2_SO_4_, and tris(hydroxymethyl)aminomethane (tris) were obtained from Sinopharm Chemical Reagent Co., Ltd (China). Thymol was supplied by E-en Chemical Technology Co., Ltd (Shanghai, China). Ethylenediaminetetraacetic acid (EDTA) was purchased from Solarbio Science & Technology Co., Ltd (Beijing, China). The Calcein-AM/PI Live-Dead Cell Staining Kit was obtained from Elabscience Biotechnology Co., Ltd (Wuhan, China). The Cell Counting Kit-8 (CCK-8) was purchased from Meilun Biotechnology Co., Ltd (Dalian, China). Phosphate-buffered saline (PBS), fetal bovine serum (FBS), penicillin-streptomycin solution, 0.25% trypsin solution, and α-minimum essential medium (α-MEM) were supplied by Procell Life Science & Technology Co., Ltd (Wuhan, China).

### 2.2 Self-assembly of peptides into PNFs

PNFs were prepared *via* a solution-induced self-assembly method. Briefly, a 1 mg mL^−1^ peptide solution was prepared in an ethanol solution containing 0.1% TFA (v/v, TFA : ethanol = 1 : 9), which was then incubated in a water-bath at 37 °C to induce the PNFs formation. The self-assembled peptide solution was subsequently used for nanostructural characterization.

### 2.3 Structural characterization of PNFs

Circular dichroism (CD) spectroscopy was applied to analyze the secondary structural features of peptide monomers and their self-assembled nanofibers. Prior to measurement, peptide samples were dissolved in the corresponding solvent system at a concentration of 2 mg mL^−1^ and thoroughly mixed. CD spectra were recorded over a wavelength range of 200–260 nm using a spectropolarimeter in a cell with a 0.1 cm path length, and baseline correction was performed with the corresponding solvent as the blank. The morphology of PNFs was analyzed by atomic force microscopy (AFM). For the sample preparation, a 10 µL of self-assembled PNFs solution was deposited onto freshly cleaved mica surfaces and allowed to dry under ambient conditions for 24 h before imaging. The peptide was dissolved in deionized water (non-assembled state) or in an ethanol/TFA mixed solvent (assembled state). After assembly, the samples were centrifuged at 10 000 rpm for 5 min, resuspended in deionized water, and examined by transmission electron microscopy (TEM) to compare the morphologies of the non-assembled, assembled, and solvent-exchanged samples.

### 2.4 Ca^2+^ titration and zeta potential measurements

The solutions of PNFs, the non-assembled full-length peptide (KLVFFAKMLPHHGA, denoted FL (non-assembled)), and MLPHHGA were prepared in ultrapure water at 0.2 mg mL^−1^. CaCl_2_ was then added to obtain Ca^2+^ concentrations of 0, 1, 2, 3, 4, and 5 mM, with each concentration prepared separately. The zeta potential was then measured. Three independent measurements were performed for each sample at each Ca^2+^ concentration.

### 2.5 Preparation of an *in vitro* demineralized dentin model

Ethical approval was obtained from the Medical Ethics Committee of the Affiliated Hospital of Qingdao University (QYFYWZLL30826), and informed consent was obtained from all donors. Human caries-free third molars were collected, cleaned, and stored in thymol solution at 4 °C prior to use. Dentin discs (1.0–1.5 mm thick) were prepared using a low-speed precision saw, with the surface opposite to the pulp chamber designated as the test surface. To standardize surface conditions, the discs were sequentially polished under wet conditions and rinsed thoroughly. Demineralization was performed by immersion in EDTA solution, followed by ultrasonic cleaning to remove residual reagents. After dehydration and drying, the surface morphology of the dentin discs was examined by SEM to evaluate the effectiveness of the demineralization treatment.

### 2.6 Surface characterization of peptide-loaded dentin discs

The pre-prepared self-assembled peptide solution was applied to the surface of demineralized dentin discs by gentle rubbing. Subsequently, dentin discs were immersed into the peptide solution to ensure complete surface coverage. The samples were then incubated in a water-bath at 37 °C for 1 h to promote peptide binding and self-assembly on the dentin surface and within dentinal tubules. After incubation, the dentin discs were gently rinsed for 5 min to remove unbound peptides, air-dried, and subsequently examined by SEM to observe the surface morphology of the peptide-loaded dentin discs. Fourier transform infrared (FTIR) spectroscopy was employed to investigate chemical structural changes in demineralized dentin before and after loading with PNFs. FTIR spectra were collected for demineralized dentin, PNFs-loaded demineralized dentin, and samples subjected to a 5 min rinsing step, in order to evaluate the interfacial interactions between PNFs and the dentin matrix as well as the wash resistance of the PNFs.

### 2.7 *In vitro* dentin remineralization and morphological analysis

Gluma desensitizer (Gluma) was selected as the positive control, and demineralized dentin discs were randomly assigned to three groups: a blank control group, a Gluma-treated group, and a PNFs-treated group. All groups were subsequently immersed in 1.5× simulated body fluid (1.5× SBF) at 37 °C. The 1.5× SBF solution was prepared by adding NaCl (12.053 g), NaHCO_3_ (0.533 g), KCl (0.338 g), K_2_HPO_4_ (0.264 g), MgCl_2_ (0.220 g), CaCl_2_ (0.438 g), and Na_2_SO_4_ (0.108 g) stepwise into 700 mL of ultrapure water. The pH was adjusted to 7.4 using tris (9.177 g) and 0.1 M HCl, and the solution volume was then brought to 1 L with ultrapure water. Throughout the mineralization period, fresh 1.5× SBF was replaced every 48 h. Samples were retrieved after 1, 3, 7, and 14 days for analysis. At each interval, dentin discs were thoroughly rinsed with ultrapure water, dehydrated *via* a graded ethanol series, and air-dried. The morphology of the newly deposited mineral layer, together with the extent of dentinal tubule occlusion, was subsequently assessed by SEM.

### 2.8 Chemical and phase characterization of remineralized products

After assembly in ethanol/TFA, the peptide was centrifuged, resuspended in deionized water, and mineralized in 1.5× SBF. Samples collected at different time points were characterized by TEM and selected area electron diffraction (SAED). Peptide-loaded demineralized dentin was mineralized in 1.5× SBF and analyzed at different time points by X-ray diffraction (XRD) after rinsing and drying. After 14 days, the samples were further examined by energy-dispersive X-ray spectroscopy (EDS), and the Ca/P atomic ratio was calculated. Crystalline characteristics of the remineralized products were examined by XRD. Diffraction data were acquired within a 2*θ* range of 10° to 70°, and phase identification was performed by comparison with the reference HAp PDF dataset (JCPDS no. 09-4032). The surface elemental composition and chemical bonding states of the remineralized products were further assessed using X-ray photoelectron spectroscopy (XPS). High-resolution spectra of the Ca 2p, P 2p, and O 1s core levels were obtained to probe the Ca–O–P bonding configuration in the mineral phase and to provide supporting evidence for an apatite-like Ca–P mineral phase.

### 2.9 Longitudinal evaluation of dentinal tubule occlusion after remineralization

After 14 days of mineralization, dentin discs were randomly selected from each group and mechanically fractured to expose the longitudinal tubule profiles. The dics were then rinsed, dehydrated, dried, and gold-sputtered prior to SEM observation to assess longitudinal dentinal tubule occlusion in each group.

### 2.10 The examination of dentin composition hardness

Vickers microhardness measurements were performed to assess dentin samples under different treatment conditions, including sound dentin, demineralized dentin, and dentin from the blank, Gluma-treated, and PNFs-treated groups after 14 days of mineralization (*n* = 6). For each disc, measurements were obtained at five distinct locations. A load of 0.5 kgf was applied with a dwell time of 10 s for each indentation. All measurements were conducted under identical experimental conditions, and the resulting hardness values were used for subsequent statistical analysis.

### 2.11 *In vitro* biocompatibility assay

Human dental pulp tissues were obtained with informed consent following approval by the Medical Ethics Committee of the Affiliated Hospital of Qingdao University (QYFYWZLL30826). Primary human dental pulp stem cells (hDPSCs) were isolated from healthy pulp tissues under sterile conditions and cultured in α-MEM complete medium at 37 °C in a humidified atmosphere with 5% CO_2_. Cells migrating from tissue explants were expanded and passage-4 hDPSCs were used for subsequent experiments. Cell identity was confirmed by flow cytometry based on the expression of mesenchymal stem cell markers CD44 and CD90 and the absence of hematopoietic markers CD34 and CD45.

For cytocompatibility evaluation, passage-4 hDPSCs were seeded in 96-well plates and treated with peptide solutions at graded concentrations (0.078–5.000 mg mL^−1^) for 1, 3, and 5 days. Cell viability and proliferation were assessed using the CCK-8 assay by measuring absorbance at 450 nm. In addition, remineralized dentin discs were co-cultured with hDPSCs in 24-well plates, and the CCK-8 assays were performed at corresponding time points to evaluate the cytocompatibility of the mineralized substrates.

The live/dead staining was further conducted to visualize cell survival. Passage-4 hDPSCs were seeded in 24-well plates, treated with peptide solutions at selected concentrations, and stained using a Calcein-AM/PI kit. Fluorescence images were acquired using a confocal laser scanning microscope, and quantitative analysis of live and dead cells was performed using ImageJ software.

### 2.12 *In vivo* biocompatibility evaluation

Healthy male Sprague–Dawley rats were used for the *in vivo* experiments. All animal procedures were conducted in accordance with relevant ethical guidelines and were approved by the Ethics Committee of the Affiliated Hospital of Qingdao University (QYFYWZLL30826). The rats were randomly divided into an experimental group and a control group. Under general anesthesia, dentin in rats from the experimental group was treated with PNFs. After treatment, the rats were maintained under standard housing conditions and euthanized after 2 weeks. Oral tissues (buccal mucosa, tongue, and palatal tissues) as well as major organs (heart, liver, spleen, lung, and kidney) were harvested for histological analysis.

### 2.13 Statistical analysis

All quantitative results are expressed as mean ± SD. Data processing and statistical analysis were carried out using GraphPad Prism (GraphPad Software, USA). Depending on the experimental design, one-way analysis of variance (ANOVA) was used for multiple-group comparisons. Statistical significance was defined as *P* < 0.05. In graphical presentations, distinct symbols denote different significance levels: *P < 0.05, **P < 0.01, ***P < 0.001, while ns indicates no statistically significant difference.

## 3 Results and discussion

### 3.1 Peptide self-assembly behaviour and Ca^2+^-responsive characteristics

**Fig. 1:**
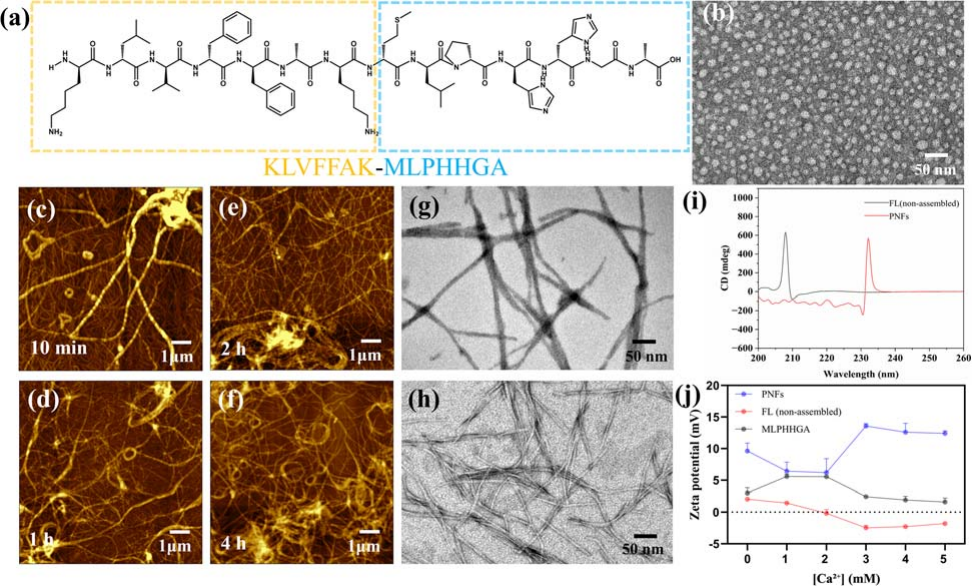
(a) Amino acid sequence of the peptide. (b) TEM image of the full-length peptide under the non-assembled condition. (c–f) AFM images of the peptide at different self-assembly times: (c) 10 min, (d) 1 h, (e) 2 h, and (f) 4 h. (g) TEM image of PNFs. (h) TEM image of PNFs after solvent exchange. (i) CD spectra of FL (non-assembled) and PNFs. (j) Zeta potential profiles during Ca^2+^ titration.

Based on its functional amino acid composition, the peptide KLVFFAK–MLPHHGA was designed to integrate a self-assembly-driving segment (KLVFFAK) with a mineralization-inducing segment (MLPHHGA), as shown in Fig. 1a. The KLVFFAK segment consists of hydrophobic residues and aromatic side chains, enabling the formation of stable fibrillar structures through β-sheet formation and hydrophobic interactions.^27,29^ TEM showed that the peptide mainly existed as dispersed particulate structures before assembly (Fig. 1b).

To further elucidate the structural features of these assemblies across different length scales, the morphology of PNFs was systematically characterized using AFM. AFM observations revealed that, after incubation in an ethanol/TFA system, the peptide rapidly underwent self-assembly, forming a large number of uniformly distributed nanoscale fibrous structures on the substrate surface (Fig. 1c–f). With increasing assembly time, the nanofiber length increased markedly, and the overall network architecture gradually became more compact. Meanwhile, inter-fiber interactions progressively intensified, with cross-linking and entanglement observed in multiple regions, driving the system to evolve from dispersed nanofibers into more complex three-dimensional (3D) architectures, characteristic of a time-dependent self-assembly process. TEM observations further confirmed the predominantly linear morphology of the formed PNFs (Fig. 1g). The nanofibers displayed well-defined boundaries, and in certain regions, individual nanofibers were found to align in parallel or associate into bundled structures. The fibrous structure remained intact after removal of the organic solvent and redispersion in deionized water, indicating good structural stability of the peptide nanofibers (Fig. 1h). CD spectroscopy revealed pronounced differences in the secondary structural features of the peptide before and after self-assembly. In the non-assembled state, the peptide exhibited a distinct positive peak at 208–210 nm, while no characteristic negative band associated with β-sheet structures was observed near 220 nm, indicating a predominantly random-coil conformation. PNFs showed a CD spectrum clearly different from that of the peptide solution, displaying a pronounced positive signal at 230–235 nm together with a negative band centered near 220 nm (Fig. 1i). These spectral features are consistent with a β-sheet-dominated secondary structure, indicating that the peptide undergoes pronounced conformational rearrangement during assembly, gradually transitioning from a relatively disordered state to an ordered nanofibrous structure enriched in β-sheet motifs. The above results demonstrate that the peptide is capable of forming well-defined nanofibers at the nanoscale and, through hierarchical assembly and reorganization, constructing a stable microscale framework, a feature comparable to that observed in other self-assembling peptide systems developed for tissue engineering and mineralization templates.^30,31^

Zeta potential measurements during Ca^2+^ titration (Fig. 1j) showed that all three samples exhibited slightly positive values in the absence of Ca^2+^, with PNFs showing the highest initial zeta potential. As the Ca^2+^ concentration increased from 0 to 5 mM, the zeta potential of PNFs remained positive overall and increased under intermediate to high Ca^2+^ conditions. By contrast, that of the FL (non-assembled) gradually decreased, approaching zero and showing a slight negative shift at high Ca^2+^ concentrations, whereas MLPHHGA exhibited only a modest change. These results indicate distinct interfacial charge responses to Ca^2+^ among peptide systems with different structural states, suggesting a potential association between peptide assembly and Ca^2+^-related interfacial processes.

### 3.2 PNF deposition on demineralized dentin surface

**Fig. 2:**
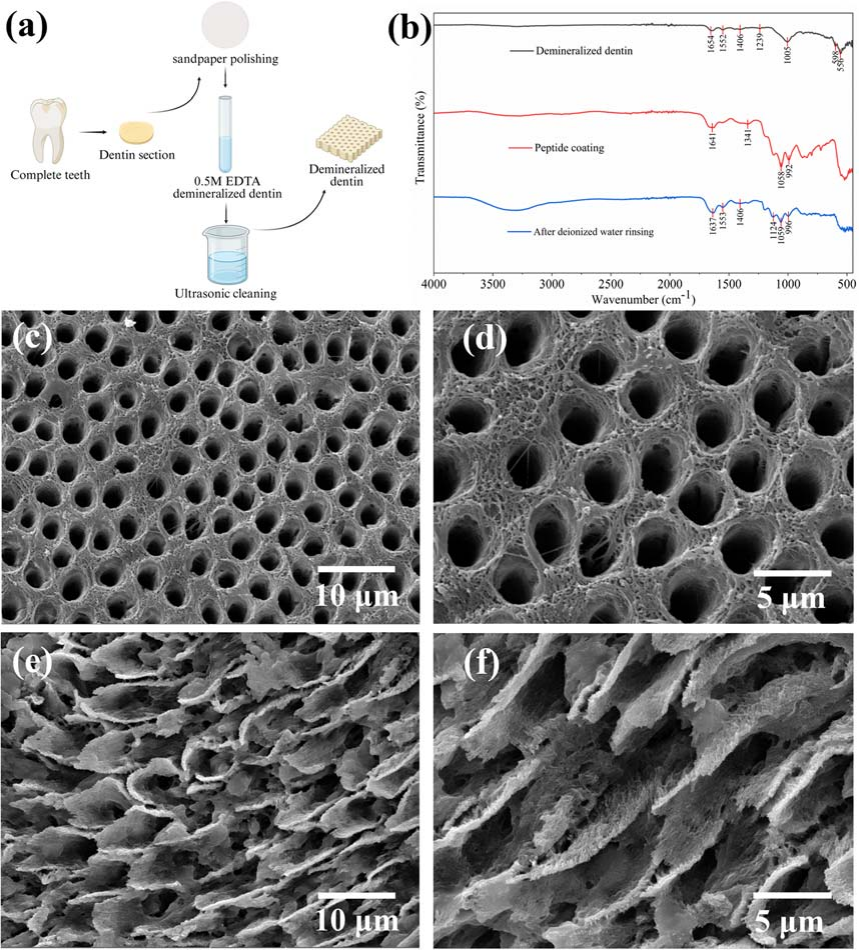
(a) Schematics of the dentin demineralization process. (b) FTIR spectra of PNFs loading. (c and d) SEM images of the surface of demineralized dentin. (e and f) SEM images of demineralized dentin after PNF loading.

As illustrated in Fig. 2a, a schematic diagram depicts the demineralization process of dentin discs, during which mineral components are removed, resulting in a characteristic demineralized structure. To characterize the loading behaviour and interfacial interactions of PNFs and demineralized dentin surfaces, FTIR was performed on demineralized dentin before and after PNFs loading, and following rinsing with ultrapure water. In parallel, SEM was employed to examine the surface morphologies of demineralized dentin and PNFs-loaded dentin, enabling evaluation of the demineralization efficacy and the surface distribution of PNFs.

To investigate the interfacial interactions between PNFs and demineralized dentin, FTIR spectroscopy was employed to analyze the relevant samples (Fig. 2b). In the FTIR spectrum of demineralized dentin, distinct absorption bands were clearly observed at approximately 1654 and 1552 cm^−1^, corresponding to the amide I band (C=O stretching vibration) and amide II band (coupled N–H bending and C–N stretching vibrations) of collagen, respectively. The preservation of these characteristic peaks indicates that the collagenous framework of dentin remained intact after the demineralization process. In addition, absorption signals near 1406 and 1239 cm^−1^ were also detected, which can be attributed to carboxylate-related vibrations and the amide III band, respectively, both of which are characteristic spectral features of dentin collagen. After PNFs loading, pronounced alterations were observed in the amide region of the FTIR spectrum. Specifically, the amide I band shifted from approximately 1654 cm^−1^ to a lower wavenumber near 1641 cm^−1^, accompanied by concurrent changes in the peak shape and intensity of the amide II band. Meanwhile, an enhanced absorption signal emerged at approximately 1341 cm^−1^, suggesting that the introduction of PNFs altered the local chemical environment of collagen amide groups. In addition, more pronounced absorption enhancements were detected in the regions around 1058 and 992 cm^−1^, which are consistent with vibrational modes associated with the peptide backbone and its related functional groups. After rinsing with deionized water, the amide I and amide II absorption bands located at approximately 1637 and 1553 cm^−1^ remained clearly discernible, with peak positions and relative intensities largely consistent with those observed prior to rinsing. Meanwhile, absorption features at approximately 1406, 1124, and 1059 cm^−1^ were still present, indicating that PNFs were still detectable on the dentin surface after rinsing. Together with the spectral changes observed before and after rinsing, these results suggest an interfacial association between PNFs and the demineralized dentin matrix under the tested conditions.

Based on these analyses, the surface morphology of demineralized dentin was further examined using SEM. As shown in Fig. 2c and d, following removal of the mineral phase, the dentinal tubule orifices were fully exposed, with collagen fibrils clearly visible and no residual mineral deposits detected on the surface. In the absence of inorganic support, the collagen matrix exhibited a typical fibrillar organization. After PNFs treatment, a large number of densely distributed nanoscale fibrous structures were observed at the tubule orifices and in the surrounding regions (Fig. 2e and f). Notably, the fibrous morphologies adhered to the dentin surface were highly similar to those of the previously self-assembled PNFs (Fig. 1), indicating that the peptide retained its self-assembly capability after anchoring to the dentin matrix.

The above results indicate that demineralized dentin exhibited completely open dentinal tubules, which not only recapitulated the microstructural features of clinically sensitive dentin but also provided abundant accessible interfaces for peptide loading. Following EDTA-induced demineralization, the collagen network became exposed, enabling peptide attachment. In addition, the peptide formed nanofibrous network, which can be regarded as an early-stage biomimetic template. Such a nanofibrous network is expected to increase the surface roughness and specific surface area, thereby enhancing local Ca^2+^/PO_4_^3−^ ion enrichment and supporting the formation of an apatite-like Ca–P phase. Previous studies have demonstrated that nanoscaffold structures formed by self-assembling peptides at mineralization interfaces can significantly promote ACP stabilization and directional HAp deposition.^6,32^ Therefore, this phenomenon provides an important foundation for achieving biomimetic remineralization with the self-assembled PNF system investigated in this study.

### 3.3 *In vitro* morphological characterization of demineralized dentin remineralization

**Fig. 3:**
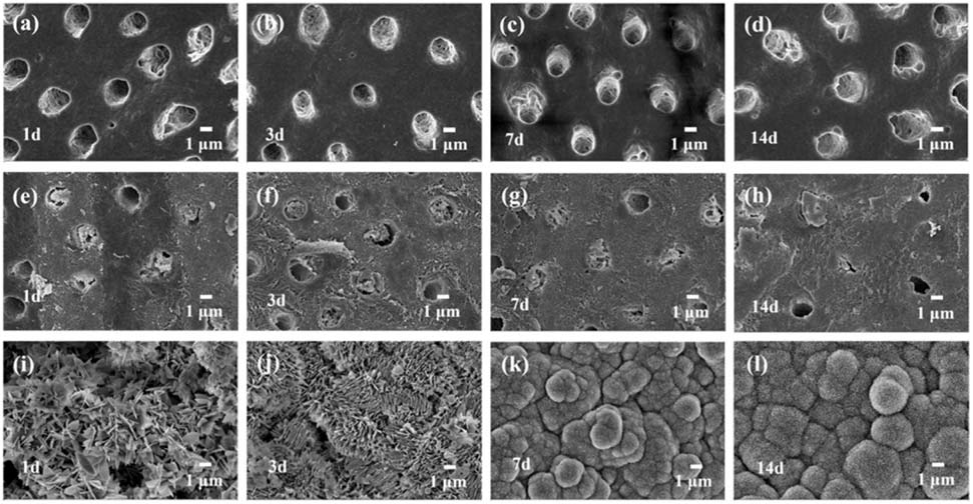
(a–d) SEM images of dentin surfaces in the blank group after mineralization in 1.5× SBF for 1, 3, 7 and 14 days. (e–h) SEM images of dentin surfaces in gluma group after mineralization in 1.5× SBF for 1, 3, 7 and 14 days. (i–l) SEM images of dentin surfaces in the PNFs group after mineralization in 1.5× SBF for 1, 3, 7 and 14 days.

To evaluate the ability of the created PNFs to promote dentin remineralization, mineralization morphologies after the incubation in 1.5× SBF with different periods (1, 3, 7, and 14 days) were examined and compared with those of the blank control and the Gluma positive control groups. Representative remineralization images are presented in Fig. 3. In the blank control group (Fig. 3a–d), the dentin surface exhibited a typical open-tubule morphology with clearly defined tubule orifices and no detectable mineral deposition, indicating that 1.5× SBF alone was insufficient to actively induce dentin mineralization in the absence of the peptide. In the Gluma positive control group (Fig. 3e–h), a moderate degree of mineral deposition was observed on the dentin surface, predominantly localized at the dentinal tubule openings and the surrounding regions. Although the amount of surface deposition increased with prolonged mineralization time, the deposits remained heterogeneously distributed, and a considerable number of dentinal tubules were still not completely occluded.

In contrast to control groups, the PNFs-treated group exhibited a pronounced time-dependent enhancement in mineralization (Fig. 3i–l). After 1 day of mineralization (Fig. 3i), numerous newly formed flake-like or needle-like deposits were observed on the dentin surface with a dense distribution, suggestive of initial mineral deposition. Together with the compositional analyses presented below, these deposits are consistent with Ca–P-based mineral formation, indicating that PNFs facilitate mineral precipitation during the early stage of mineralization. After 3 days of mineralization (Fig. 3j), the initial deposits evolved into abundant platelet-/leaf-like mineral structures on the surface. These features exhibited extensive lateral overlap and, in some regions, formed interconnected lamellar assemblies, which is suggestive of progressive mineral maturation and increased structural ordering. With prolonged mineralization up to 7 days (Fig. 3k), further growth and gradual coalescence of the mineral deposits were observed, leading to the formation of a largely continuous mineralized layer on the dentin surface. At this stage, pronounced surface protrusions became evident, accompanied by increased structural continuity and compactness, indicating that the mineralization process had entered a relatively stable growth phase.^22^ When the mineralization period was extended to 14 days (Fig. 3l), the dentin surface was uniformly covered by a dense and continuous mineral layer, resulting in the formation of a structurally complete and well-developed mineralized coating. The crystals exhibited extensive intercrystalline fusion, giving rise to characteristic microscale mound-like mineralized structures. This highly integrated surface coverage indicates that, during prolonged mineralization, the peptide may facilitate continued apatite-like mineral deposition and structural consolidation, ultimately resulting in a stable biomimetic mineralized layer.

### 3.4 Morphological and phase evolution of remineralization products

**Fig. 4:**
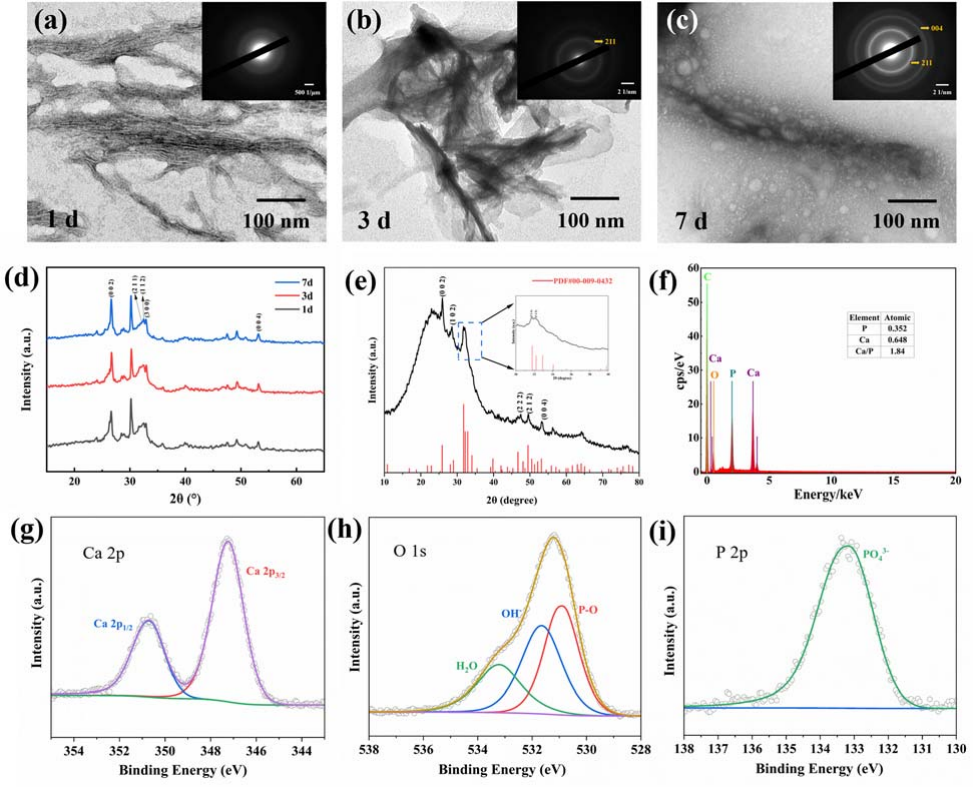
(a–c) TEM images and SAED patterns of mineral deposits at 1, 3, and 7 days. (d) XRD patterns of the mineralized samples at 1, 3, and 7 days. (e) XRD pattern of the 14 day mineralized sample. (f) EDS spectrum of the 14 day mineralized sample. (g–i) XPS spectra of the 14 day mineralized sample.

To clarify the phase composition of the mineralized products and their temporal evolution, samples collected after 1, 3, and 7 days of PNFs-induced mineralization were examined by TEM and SAED, and the corresponding XRD patterns were recorded. After 1 day, TEM mainly showed low-contrast deposits associated with the fibrous structures, while the SAED pattern was dominated by diffuse scattering (Fig. 4a), suggesting amorphous-like or poorly ordered Ca–P features in the early deposits. By day 3, the deposits exhibited increased electron density, and discernible diffraction rings began to appear in the SAED pattern (Fig. 4b), indicating increased structural order. By day 7, clearer polycrystalline rings were observed (Fig. 4c), consistent with apatite-like calcium phosphate minerals, indicating further enhanced crystallinity and a trend toward apatite-like mineralization.

The XRD results were consistent with the TEM observations, suggesting that the apatite-like mineral phase showed a trend toward increased structural order and crystallinity with prolonged mineralization time (Fig. 4d). The XRD pattern of the remineralization products (Fig. 4e) exhibited diffraction peaks at the (002), (211), and (112) planes, which are well consistent with characteristic diffraction peaks of apatite-like calcium phosphate, in agreement with previous reports.^21,33^ In addition, the diffraction peaks were relatively broad with low intensities, indicative of a low-crystallinity apatite-like phase, which shares similarities with the biological apatite present in natural dentin and bone tissues.^34^ This result suggests that peptide-induced mineralization yields products with biomimetic characteristics resembling those of biological mineralization. Further EDS analysis at day 14 revealed distinct Ca and P signals, with a Ca/P atomic ratio of approximately 1.84 (Fig. 4f). This value was slightly higher than that of stoichiometric hydroxyapatite (1.67), suggesting the formation of a non-stoichiometric, Ca-rich apatite-like mineral.

XPS analysis (Fig. 4g–i) revealed distinct Ca, P, and O elemental signals in the remineralized samples. The Ca 2p spectrum exhibited a typical spin–orbit doublet consisting of Ca 2p_3/2_ and Ca 2p_1/2_ peaks, with binding energies characteristic of Ca^2+^. The P 2p spectrum appeared as an overlapped envelope composed of P 2p_3/2_ and P 2p_1/2_ components, corresponding to PO_4_^3−^. In the O 1s spectrum, in addition to oxygen associated with PO_4_^3−^, a component attributed to hydroxyl groups (–OH) was also observed.^35^ Collectively, XPS results suggested that the peptide-induced mineralized products are predominantly composed of an apatite-like Ca–P phase, with a chemical composition sharing similarities with that of the mineral phase in natural dentin.

Together with the XRD results, these features suggest that that the peptide-induced mineralized products are primarily apatite-like Ca–P and share substantial similarity with the inorganic components of natural dentin, thereby supporting the biomimetic mineralization potential of the peptide.

TFA, a common counterion in synthetic peptides, has been reported to influence the physicochemical properties and experimental behavior of peptides.^36^ therefore, the potential effect of residual TFA should be considered. Because apatite-like mineral formation in 1.5× SBF is sensitive to solution conditions, substantial acidification caused by residual TFA could potentially affect mineralization kinetics.^37^ However, the progressive formation of apatite-like deposits was still observed over time, suggesting that residual TFA was unlikely to dominate the mineralization environment under the present experimental conditions. Nevertheless, because residual TFA was not quantitatively measured in this study, its specific effects cannot be fully excluded.

### 3.5 Evaluation of dentin mineralization depth and microhardness

**Fig. 5:**
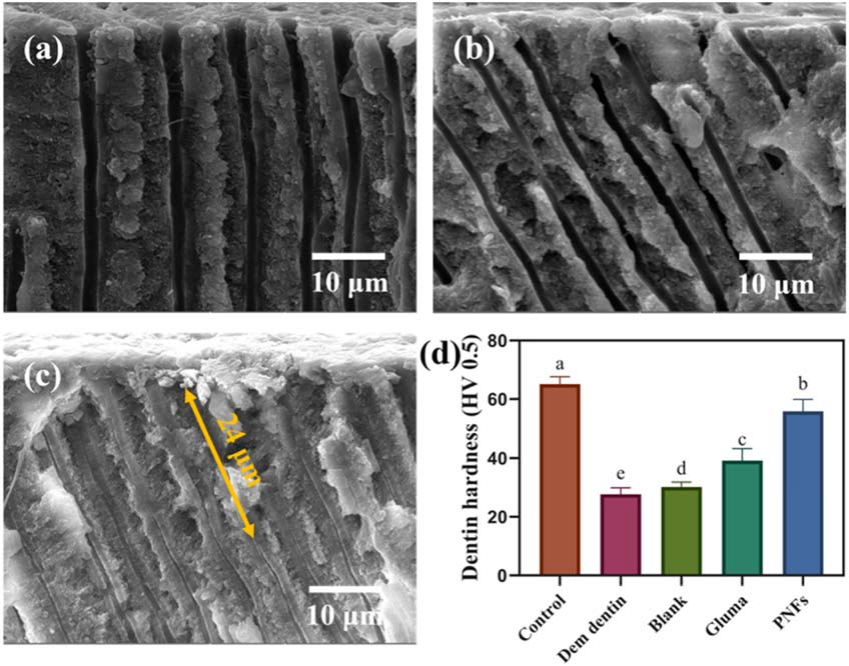
Dentinal tubule morphology of dentin samples from different groups after 14 days of treatment: (a) blank group; (b) Gluma group; (c) PNFs group. (d) Vickers microhardness results. Different letters indicate statistically significant differences (*p* < 0.05).

In blank group (Fig. 5a), the dentinal tubules retained a typical hollow morphology with no evident mineral deposition. The tubule inner walls appeared smooth and continuous, indicating that spontaneous intratubular mineralization was unlikely to occur in the absence of an inducing factor. Observation of the Gluma-treated dentin discs (Fig. 5b) revealed that tubule occlusion occurred predominantly at the tubule orifices, where a relatively thin occlusive layer was formed, whereas little to no mineral deposition was observed within the deeper tubule lumen or along the inner tubule walls. These morphological features indicate that the effect of Gluma is mainly limited to surface-level occlusion, with a minimal influence on mineral formation within the dentinal tubules. Dentin treated with PNFs exhibited mineralization morphologies that were markedly different from those observed in the control group (Fig. 5c). Mineral deposition was no longer confined to the tubule orifices but extended axially along the dentinal tubules, with the tubule lumen appearing to be continuously filled by mineralized deposits and progressively narrowed. The intratubular mineral penetration depth after PNFs treatment was quantified based on 8 cross-sectional SEM images from 4 independent tooth specimens (45 tubules in total), yielding a mean value of 16.90 ± 5.03 µm with a maximum of 24.43 µm. These SEM observations indicate that the role of PNFs is not limited to surface tubule occlusion but also involves promoting mineral growth and deposition within the dentinal tubules.

Vickers microhardness measurements (Fig. 5d) demonstrated that EDTA-induced demineralization resulted in a pronounced reduction in dentin hardness, whereas only limited hardness recovery was observed in the blank and Gluma-treated groups. In contrast, dentin treated with PNFs exhibited a markedly greater increase in hardness, with microhardness values approaching those of sound dentin. Taken together with the morphological observations, these results suggest a consistency in trend between the extensive and continuous intratubular mineralization and the improvement in surface microhardness. However, given the limited scope of mechanical evaluation, the specific contribution of deep mineral deposition to dentin mechanical recovery requires further verification.

### 3.6 Biocompatibility of PNFs

**Fig. 6:**
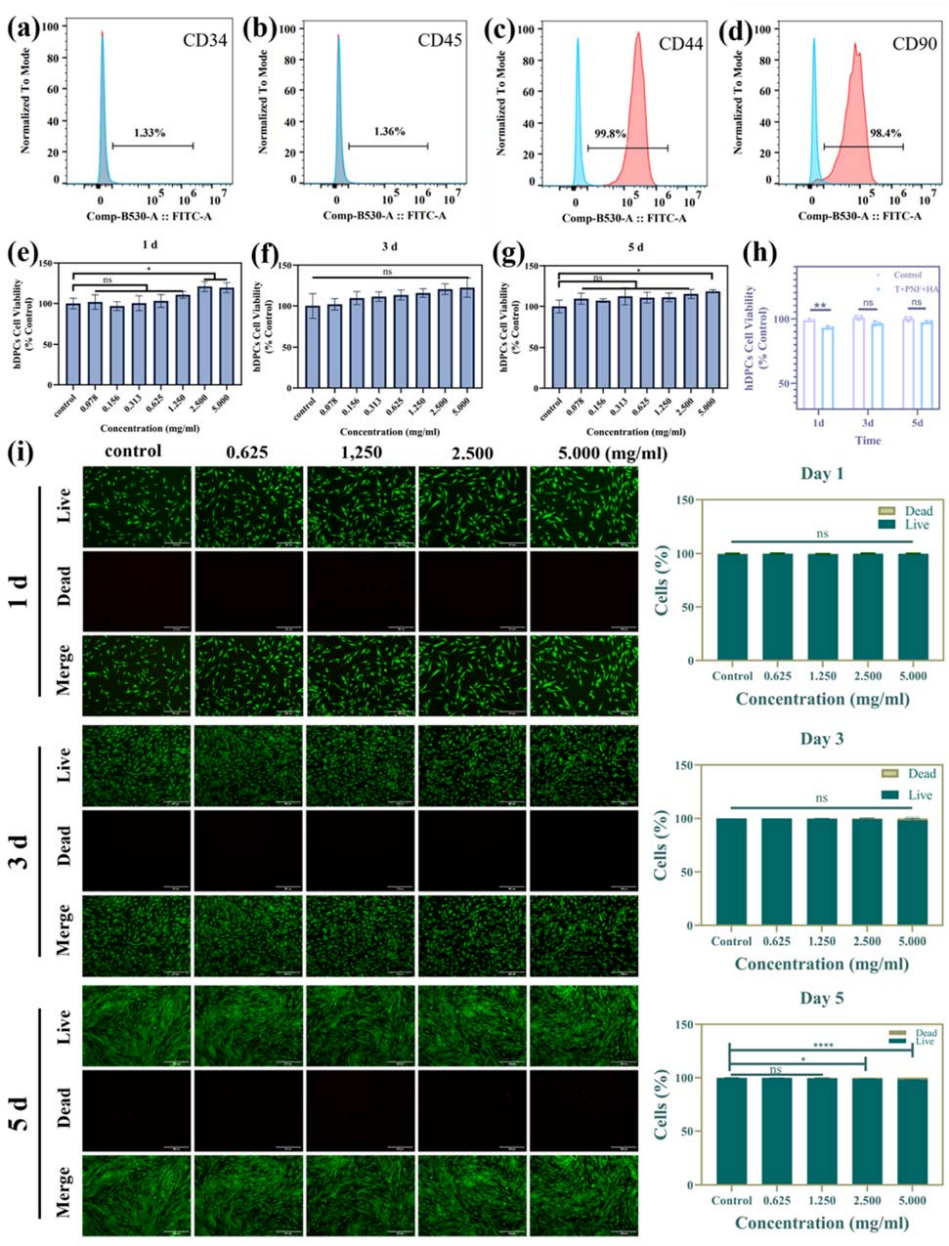
(a–d) Flow cytometric analysis for cell phenotype identification: (a) CD34, (b) CD45, (c) CD44, and (d) CD90. (e–g) CCK-8 assay results of the peptide-treated group after 1, 3 and 5 days. (h) CCK-8 assay results of the dentin remineralization group after 1, 3 and 5 days. (i) CLSM images of live/dead cell staining and the corresponding percentages of live and dead cells.

To confirm the identity of the isolated cells, flow cytometric analysis was performed on the cell population derived from dental pulp tissue (Fig. 6a–d). The results demonstrated that the vast majority of cells exhibited strong positive expression of mesenchymal stem cell–associated surface markers CD44 and CD90, with positivity rates exceeding 95%, whereas the expression of hematopoietic markers CD34 and CD45 was below 2%. This marker expression profile is highly consistent with the established immunophenotype of hDPSCs, indicating that the obtained cell population exhibits good homogeneity and is suitable as a reliable cellular model for subsequent biocompatibility assessments.

After confirming the cellular phenotype, the CCK-8 assay was further employed to evaluate the biological effects of the peptide on cells. The results showed that cell viability in the peptide-treated groups remained comparable to that of the control group over culture periods of 1, 3, and 5 days (Fig. 6e–g). As the culture period progressed, the absorbance at 450 nm increased gradually, indicating sustained cell proliferation. Although minor fluctuations were observed among the different concentration groups, no statistically significant differences were detected. Cells exposed to the remineralization products also exhibited metabolic activity comparable to that of the control group (Fig. 6h). These results suggest that neither the peptide nor the associated mineralization products exert adverse effects on the viability of hDPSCs.

To further assess the cell viability and morphological features, the live/dead staining test was performed on the cultured cells (Fig. 6i). Under peptide-treated conditions, the majority of cells exhibited strong green fluorescence corresponding to Calcein-AM staining, whereas PI-positive cells were rarely observed. At the same time, the cells maintained a well-defined spindle-like morphology comparable to that of the control group, with no evident signs of membrane damage or abnormal structural changes. Taken together with the preceding results, these observations indicate that the peptide demonstrates favorable cytosafety.

**Fig. 7:**
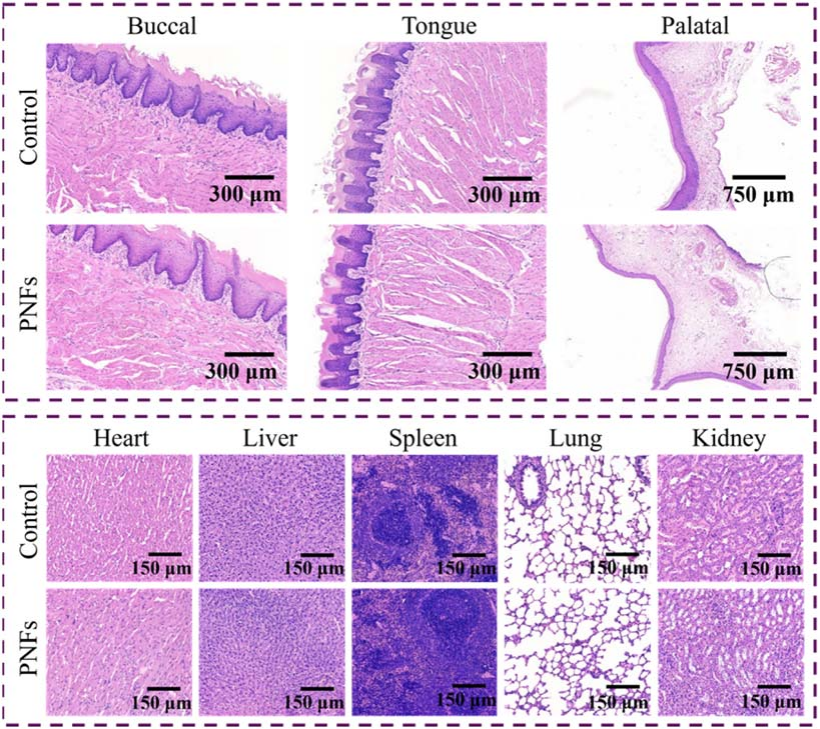
H&E-stained images of oral mucosal tissues and major organs.

Histological evaluation was performed on the relevant tissues using hematoxylin–eosin (H&E) staining (Fig. 7). Compared with the control group, no discernible structural abnormalities or pathological alterations were observed in the oral mucosal tissues of rats, including the buccal mucosa, tongue, and palate, nor in major organs such as the heart, liver, spleen, lung, and kidney. These histological findings indicate that the PNFs-based system exhibits favorable *in vivo* biocompatibility.

## 4 Conclusions

By introducing a short peptide capable of both mineralization induction and fibrous self-assembly, this study established a biomimetic mineralization system that enabled the remineralization of demineralized dentin. Experimental observations showed that, under appropriate conditions, the peptide could spontaneously assemble into a collagen-like nanofibrous network and remain detectable on the demineralized dentin surface, thereby providing a continuous organic framework. When exposed to 1.5× SBF, calcium–phosphate minerals evolved toward an apatite-like Ca–P phase and formed continuous mineralized structures extending into the dentin. In parallel, the treatment with PNFs resulted in a pronounced improvement in dentin microhardness. Moreover, both *in vitro* and *in vivo* biocompatibility evaluations revealed no evident adverse effects, indicating that the PNFs system possesses favorable biosafety. These findings shown in this study suggest that a self-assembling PNFs-mediated biomimetic mineralization strategy offers a viable materials design approach for dentin repair, combining structural biomimicry with functional restoration potential and holding promise for future clinical translation in dentin tissue engineering.

## Conflicts of interest

There are no conflicts to declare.

## Data Availability

The data that supports the finding of this study are available within the article.
